# Elevated serum matrix metalloprotease (MMP-2) as a candidate biomarker for stable COPD

**DOI:** 10.1186/s12890-020-01323-3

**Published:** 2020-11-16

**Authors:** Durga Mahor, Vandana Kumari, Kapil Vashisht, Ruma Galgalekar, Ravindra M. Samarth, Pradyumna K. Mishra, Nalok Banerjee, Rajnikant Dixit, Rohit Saluja, Sajal De, Kailash C. Pandey

**Affiliations:** 1 ICMR-National Institute for Research in Environmental Health, Bhopal, India; 2 ICMR-National Institute of Malaria Research, New Delhi, India; 3 Bhopal Memorial Hospital & Research Centre, Bhopal, India; 4 All India Institute of Medical Sciences, Bibinagar, Hyderabad India; 5 All India Institute of Medical Sciences, Bhopal, India

**Keywords:** Biomarker, COPD, Serine proteases, Cysteine proteases, Metallo proteases, Caspases, Protease inhibitors

## Abstract

**Background:**

The increasing trend of Chronic Obstructive Pulmonary Disease (COPD) in becoming the third leading cause of deaths by 2020 is of great concern, globally as well as in India. Dysregulation of protease/anti-protease balance in COPD has been reported to cause tissue destruction, inflammation and airway remodelling; which are peculiar characteristics of COPD. Therefore, it is imperative to explore various serum proteases involved in COPD pathogenesis, as candidate biomarkers. COPD and Asthma often have overlapping symptoms and therefore involvement of certain proteases in their pathogenesis would render accurate diagnosis of COPD to be difficult.

**Methods:**

Serum samples from controls, COPD and Asthma patients were collected after requisite institutional ethics committee approvals. The preliminary analysis qualitatively and quantitatively analyzed various serum proteases by ELISA and mass spectrometry techniques. In order to identify a distinct biomarker of COPD, serum neutrophil elastase (NE) and matrix metalloprotease-2 (MMP-2) from COPD and Asthma patients were compared; as these proteases tend to have overlapping activities in both the diseases. A quantitative analysis of the reactive oxygen species (ROS) in the serum of controls and COPD patients was also performed. Statistical analysis for estimation of *p*-values was performed using unpaired t-test with 95% confidence interval.

**Results:**

Amongst the significantly elevated proteases in COPD patients vs the controls- neutrophil elastase (NE) [*P < 0.0241*], caspase-7 [*P < 0.0001*] and matrix metalloprotease-2 (MMP-2) [*P < 0.0001*] were observed, along with increased levels of reactive oxygen species (ROS) [*P < 0.0001*]. The serum dipeptidyl peptidase-IV (DPP-IV) [*P < 0.0010*) concentration was found to be decreased in COPD patients as compared to controls. Interestingly, a distinct elevation of MMP-2 was observed only in COPD patients, but not in Asthma, as compared to controls. Mass spectrometry analysis further identified significant alterations (fold-change) in various proteases (carboxy peptidase, MMP-2 and human leukocyte elastase), anti-proteases (Preg. zone protein, α-2 macroglobulin, peptidase inhibitor) and signalling mediators (cytokine suppressor- SOCS-3).

**Conclusion:**

The preliminary study of various serum proteases in stable COPD patients distinctly identified elevated MMP-2 as a candidate biomarker for COPD, subject to its validation in large cohort studies.

## Background

Chronic obstructive pulmonary disease (COPD) is a common, preventable & treatable disease, characterized by the irreversible & progressive airflow obstruction in the lungs, usually due to the exposure to noxious particles or gases. COPD is the most significant chronic respiratory disease with high mortality rates, globally, as well as in India. Approximately, 55.3 million cases of COPD were reported in 2016 from India, with an increase in prevalence from ~ 3.3% (1990) to ~ 4.2% (2016, 1). COPD has been estimated to be responsible for 75.6% disability-adjusted life-years (DALYs) of all the chronic respiratory diseases in the Indian context [1]. Globally, COPD has been projected to be the third leading cause of deaths by 2020 (GOLD report, 2019), with inevitable increase in future due to the aging population and continued exposure to the COPD risk factors (air pollution, tobacco smoke etc.). Tobacco smoke is the most common risk factor for COPD, in addition to the other factors such as indoor air pollution, occupational exposures, host genetic factors, age & sex, lung growth & development and socioeconomic status [2].

In molecular context, COPD pathogenesis is reminiscent of tissue destruction factors, inflammation, airway remodelling and the accompanying pathways/mediators [3]. The characteristic emphysema in COPD could be attributed to the dysreguatlion of the protease/anti-protease balance [4, 5]. Various classes of proteases (serine, cysteine and metallo proteases) have previously been reported in the pathogenesis of COPD [4]. Notably, the serine protease- neutrophil elastase (NE) has been shown to play a crucial role in the destruction of alveolar tissue and development of emphysema [6]. Precise regulation of NE activity is regulated by its inhibitor- A1AT (α-1 antitrypsin) [7] and its genetic deficiency (A1AT) has been reported to predispose an individual to early onset of emphysema [8]. Another serine exopeptidase- dipeptidyl peptidase-IV (DPP-IV) is crucial in regulating the inflammatory responses in the lungs by antagonising various inflammatory cytokines. Hence, a significant decrease in DPP-IV levels in COPD patients has been concluded as a good serological marker of COPD [9]. The role of cysteine proteases in the pathogensis of COPD has been established through destruction of alveolar epithelial and endothelial cells via proteolytic activities of caspases- [3/8/9] [10]. Degradation of the extracellular matrix is a characteristic feature of COPD, causing emphysema, which is accomp;lished by various matrix metalloproteinases (MMPs)- [9/12/13] [11–15].

In this preliminary study, we set out to explore various serum proteases involved in the pathogeneis sof COPD. The goal of the study was to evaluate serum protease, as candidate serological biomarkers for stable COPD. We performed qualitative and quantitative measurements of various serum proteases in stable COPD patients and controls. Selected protease with overlapping activities in Asthma were also compared for any distinctive elevation. Further, proteome analysis via mas spectrometry of the sera from stable COPD pateints and control was also attempted.

## Methods

### Sample collection

The inclusion criteria for stable COPD patients was- symptoms (dyspnoea, chronic cough/sputum); exposure to risk factors such as smoking; ratio (FEV_1_:FVC) < 0.7 in the spirometry test and no exacerbation during the last 3 months. COPD patients with active pulmonary tuberculosis, heart diseases, kidney diseases and cancer were excluded from the study, which could interfere with the expression profile of various serum proteases. 10 asthmatic patients were enrolled in this study by following the Global Initiative for Asthma (GINA) 2018 guidelines. Written informed consent was obtained from all the patients before sample collection. 3 ml of venous blood was collected from (*n* = 35) COPD patients and (*n* = 15) controls under aseptic conditions. The blood samples were allowed to clot by leaving it undisturbed at room temperature and centrifuged at 2000×g for 1 min.; serum was collected, aliquoted and stored at − 80 °C till further use. For comparative analysis of specific proteases, a total of (*n* = 10) asthmatic patients were also enrolled in this study by following the Global Initiative for Asthma (GINA) 2018 guidelines.

### Qualitative measurement of serum NE, DPP-IV, caspases and MMPs

Direct-Enzyme-Linked Immunosorbent Assay (ELISA) was performed to measure the serum proteases- (NE, DPP-IV, caspases- [3 & 7] and MMP- [2 & 9]; in different COPD patients and the controls. The sera samples were diluted (1:100) in 1X PBS and coated in 96-well plates; incubated overnight at 4 °C. After coating of the antigen, well contents were aspirated and blocking buffer (0.5% BSA in 1X PBS) was added to each well, followed by incubation for 2 h at 25 °C. Primary antibodies [anti-elastase (1:1000); anti-caspase- [3 & 7] (1:2000)] and anti-MMP- [2 & 9] (11000)], were added and incubated for 2 h. at room temperature. After primary antibody incubation, the plates were thoroughly washed with wash buffer (0.05% Tween-20 in 1X PBS) and secondary antibody- anti-mice HRP (13000) (Santa Cruz Biotechnology) was added and plates were again washed (three times) and the peroxidase substrate solution (*o*-Phenylenediamine dihydrochloride (OPD)) in sodium citrate, pH -5.0 + H_2_O_2_) was added. As the peroxidase reacted with the OPD, a dark yellow product was formed; the intensity of the yellow colour was proportional to the amount of tested antigens in the sera samples. Stop solution was added to terminate the reaction followed by 30 min incubation and absorbance was recorded at 405 nm by using an ELISA plate reader.

### Fluorometric assays for human Caspase-3/7 activity

The activity of human caspases-3/7 in COPD patients and the controls sera were assessed by measuring the cleavage of a fluorogenic substrate [Z-DEVD-AMC] at excitation and emission wavelengths of 355 nm & 460 nm, respectively. Assay was performed using HEPES buffer (50 mM HEPES pH 7.5, 150 mM NaCl & 5 mM DTT). Purified recombinant human caspase-[3 & 7] were used as controls. All reactions were performed in duplicate and data was analysed using Graphpad Prism 5.0.

### Recombinant expression and purification of MMP-2

The recombinant construct of MMP-2 was gifted by Raquel Gerlach, University of Sao Paulo, Ribeirao Preto, SP, Brazil and expressed as per the protocol described earlier [16]. Briefly, the recombinant construct was transformed into BL21(DE3)/pLysS *E. coli* cells. Single colonies were grown in LB media containing 100 μg/mL ampicillin and 34 μg/mL chloramphenicol and 20% glucose. The culture was allowed to grow overnight at 37 °C with shaking at 180 rpm. Further, secondary culture (500 ml) was inoculated with 1% of overnight grown culture, with appropriate antibiotics and grown at 37 °C until an OD 600 of 0.5–0.7 was reached. The secondary was induced with 0.5–1 mM IPTG and allowed to grow further for 18 h at 18^0^ C. Cells were then harvested and resuspended into the phosphate buffer for lysis by sonication followed by centrifugation at 14000 RPM at 4 °C for 15–20 min. The supernatant was collected and purified by NI-NTA affinity chromatography using imidazole gradient. The purified fractions of protein was analysed by SDS-PAGE.

### Quantitative estimation of DPP-IV, NE and MMP-2 in COPD patients and controls

The quantiation of DPP-IV was performed using DPP-IV human ELISA kit (Thermo Fisher Scientific), as per the manufacturer’s protocol. To estimate the concentration of serum NE and MMP-2 in COPD patients and the controls, recombinant NE and MMP-2 (1 mg/ml stock) were coated in 96-well plates with a range of (1.25–800 μg) protein per well. Direct ELISA was performed to generate a standard curve for the above proteins. The respective proteins were estimated by standard curves.

### Measurement of free radicals

Intracellular reactive oxygen species (ROS levels) was measured in COPD patients and the controls sera. The sera samples were diluted (1:100) in 1X PBS followed by incubation with a cell-permeant dye [1X of H2DCFDA (dichloro-dihydrofluorescein diacetate) (Sigma)] for 30 min. in a 96-well plate. Fluorometric measurements (excitation at 510 nm and emission at 530 nm) were performed in duplicate, and the results were expressed as the mean fluorescence intensity.

### Mass spectrometry analysis of COPD patients and controls

Proteomics analysis of stable COPD patients and control sera samples was peformed by resolving diluted sera samples (1:20) in 15% SDS-PAGE (20 × 18.3 cm). Protein bands with different expression were selected from SDS-PAGE and preserved for identifying the protein sequence identity. Mass spectrometry was performed at Central Instrumental facility (CIF) South campus, University of Delhi, India.

## Results

### Neutrophil elastase and DPP-IV (serine proteases)

**Fig. 1:**
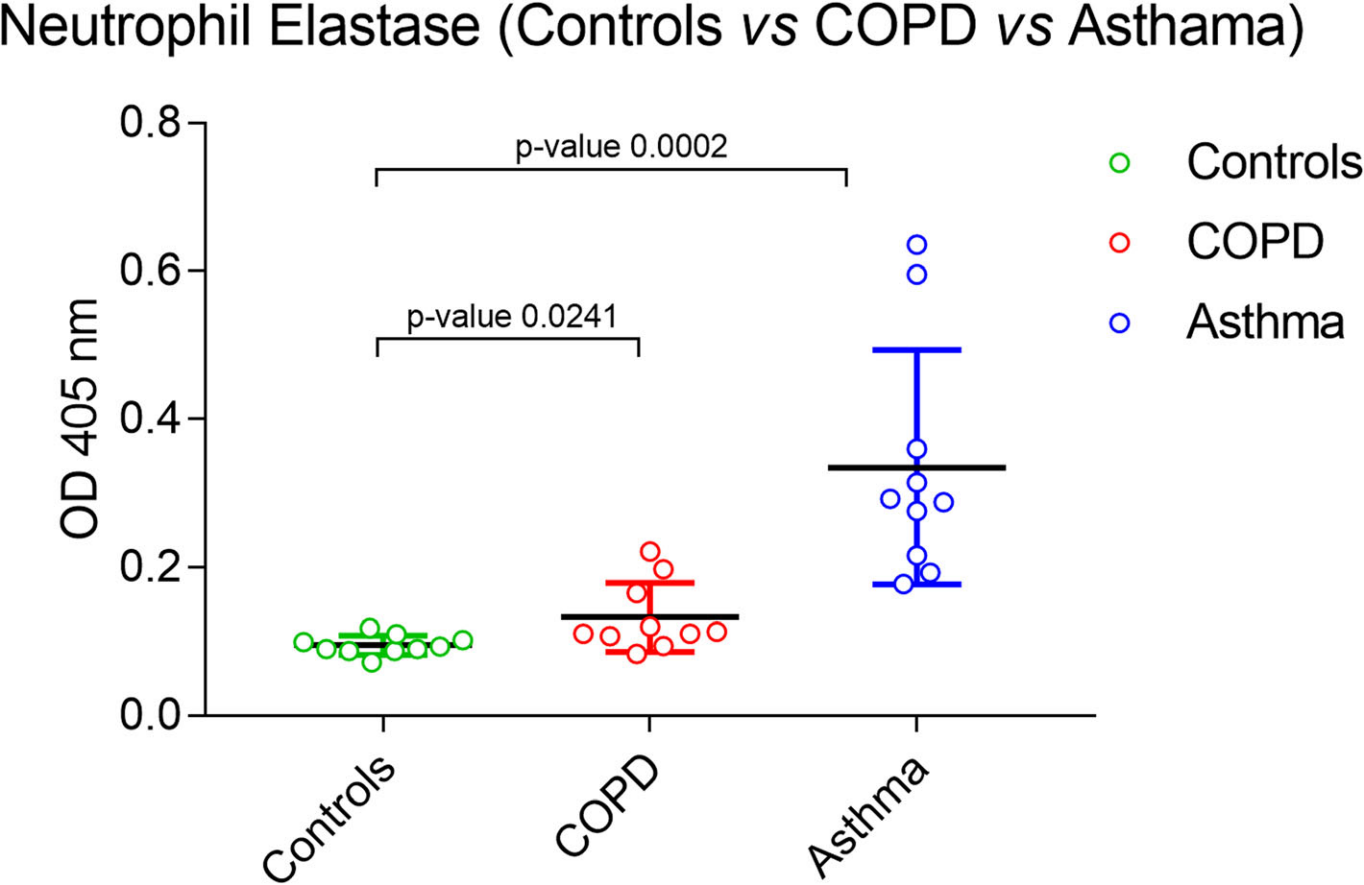
Qualitative analysis of serum NE from controls vs COPD vs Asthma patients. The *p*-values are calculated by unpaired t-test with 95% confidence interval using Graphpad prism 5.0 software

NE has been repeatedly implicated in the pathogenesis of COPD due to its potential role in the development of emphysema by degrading the extracellular matrix in the lungs [17]. Elevated NE in sputum of Asthma patients and its role in hypersecretion from goblet cells, have been reported in previous studies [18, 19]. Asthma is another impotant inflammatory respiratory disease and its symptoms often overlap with COPD such as coughing, wheezing and shortness of breath. Therefore, any specific biomarker for COPD should be able to differentiate Asthma from COPD. In this study, we performed qualitative analysis of serum NE from equal number of subjects from three groups- controls, COPD and Asthma patients. The qualitative analysis revealed a less profound difference between serum NE from controls and COPD patients [*p-0.0241*; 95% CI] as compared to a significant elevation in serum NE between controls and Asthma patients [*p = 0.0002*; 95% CI] (Fig. 1). Further, the quantitative analysis of serum NE in COPD patients estimated average concentration of (0.21 ± 0.018 μg/ml) as compared to controls (0.047 ± 0.014 μg/ml), represented in Table 1.

**Table 1:** Quantitative analysis of serum NE and MMP-2 in COPD patients vs the controls

	Neutrophil elastase (μg/ml)	Matrix metalloprotease-2 (μg/ml)
Controls	0.047 ± 0.014	0.05 ± 0.0083
COPD patients	**0.21 ± 0.018**	**0.71 ± 0.0647**

**Fig. 2:**
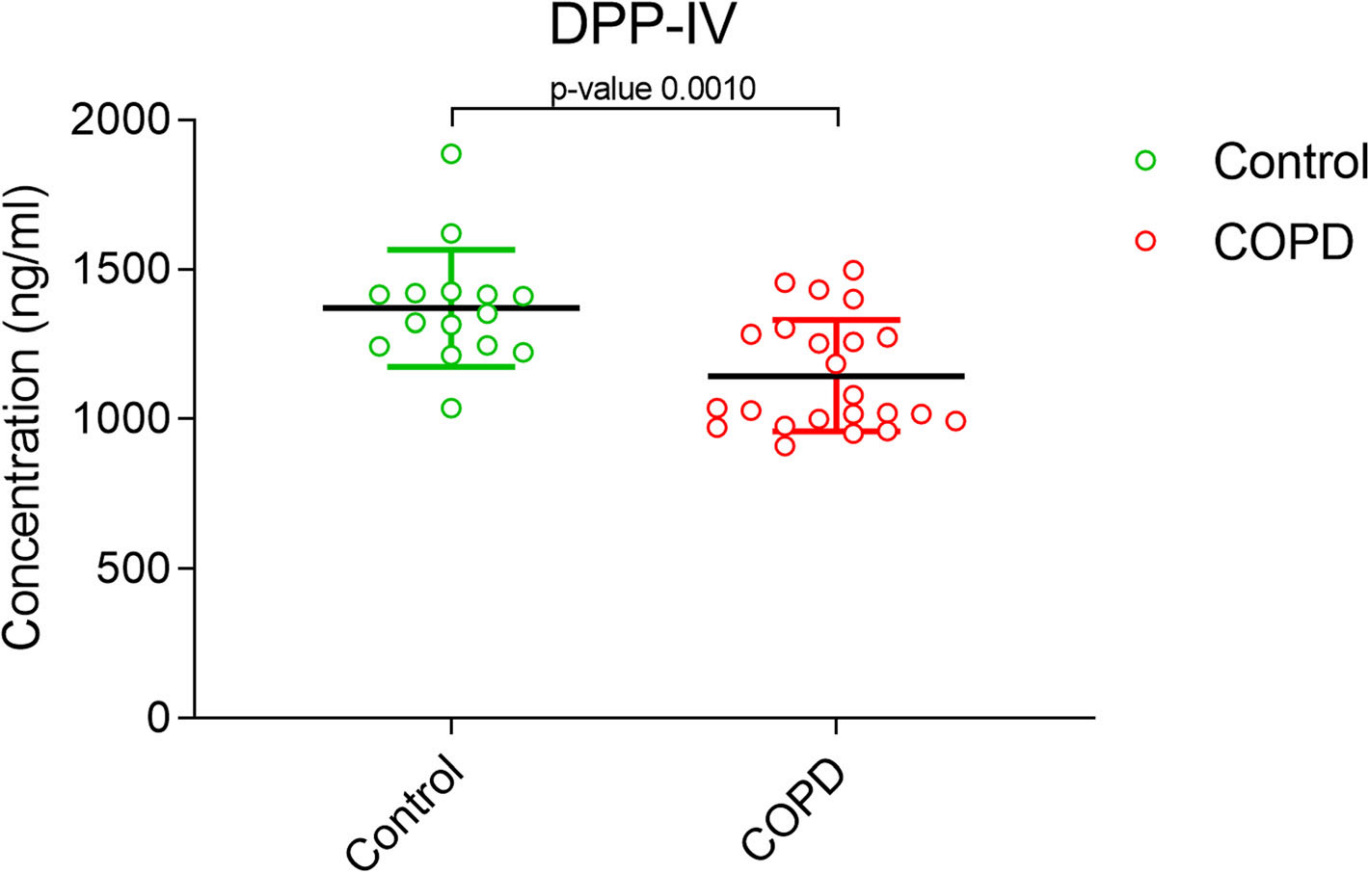
Measurement of serum DPP-IV levels in COPD patients vs the controls. The p-values are calculated by unpaired t-test with 95% confidence interval using Graphpad prism 5.0 software

DPP-IV or CD26 is antoher serine exopeptidase, which has recently been reported to have significantly lower concentration in COPD patients [9]. The decreased activity of the soluble DPP-IV has been shown to be an indicator of COPD [20]. However, a less profound decrease in serum concentration of DPP-IV in COPD patients as compared to the controls [*p = 0.0010*; 95% CI] (Fig. 2) was observed in our study. Quantitative analysis estimated a range of (1200–1800 ng/ml) in controls group as compared to COPD patients (900–1100 ng/ml).

### Caspases- [3 & 7] (cysteine proteases)

**Fig. 3:**
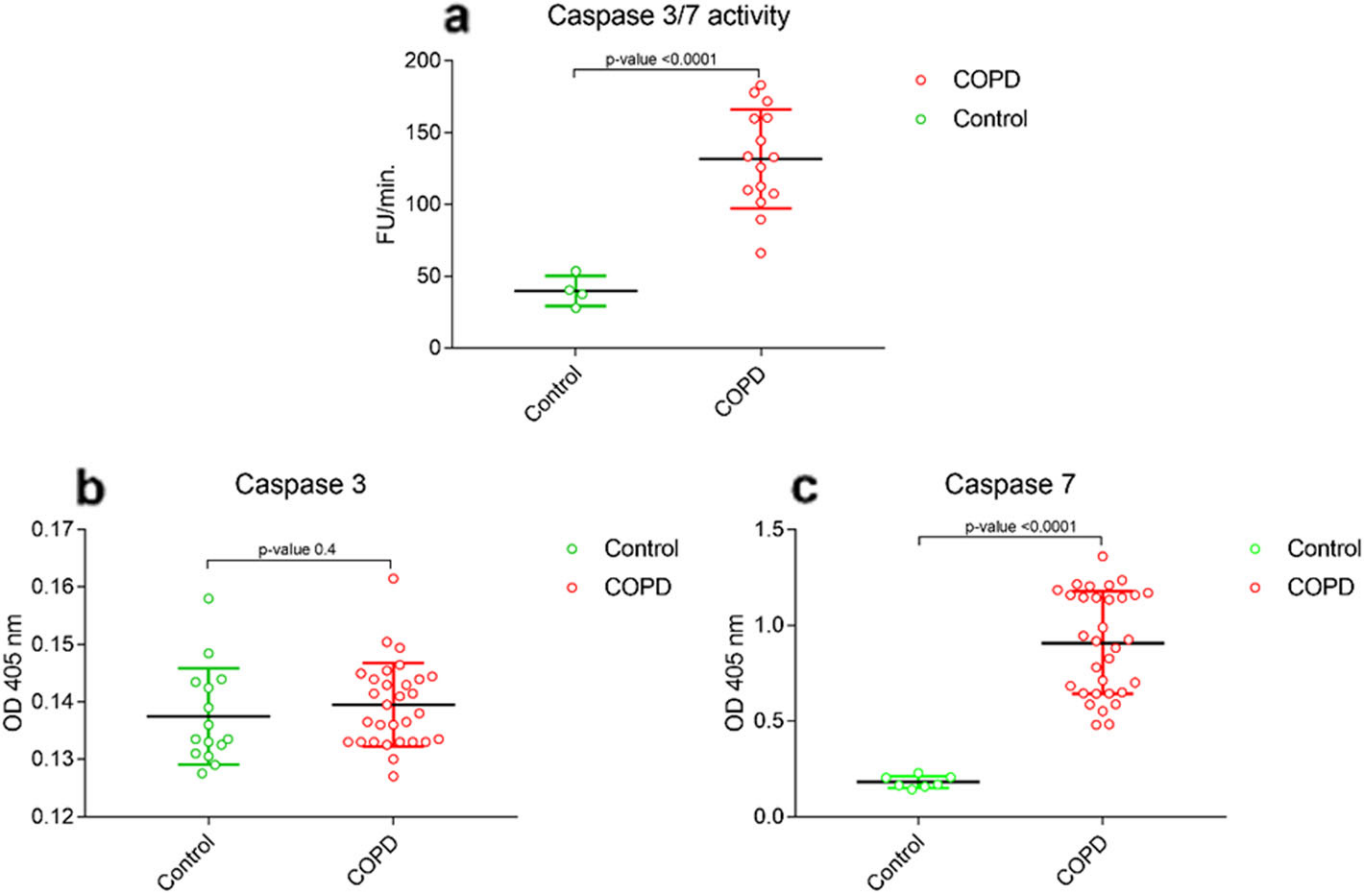
Activity measurement and qualitative analysis of serum caspases- [3&7]. (**a**) estimation of caspase-3/7 activity; (**b**) qualitative estimation of serum caspase-3; and (**c**) caspase-7 from controls vs COPD patients. The p-values are calculated by unpaired t-test with 95% confidence interval using Graphpad prism 5.0 software

Different caspases have been shown to be the mediators of apoptotic processes in COPD, with probable activation by the extracellular signals or intrinsic pathways (mitochondrial and endoplasmic reticulum) [10]. An approximate 3-fold higher caspase- 3/7 activity was observed in COPD patients vs controls [*p < 0.0001*; 95% CI] (Fig. 3a). Further, the qualitative analysis of caspase- [3&7] in the sera samples of controls and COPD patients was performed. The serum caspase-3 was not found to be significantly different in COPD patients vs controls [*p = 0.04*; 95% CI] (Fig. 3b). However, a significant elevation in serum caspase-7 was observed in CODP patients as compared to controls [*p < 0.0001*; 95% CI] (Fig. 3c).

### MMP- [2 & 9] (matrix metalloproteases)

**Fig. 4:**
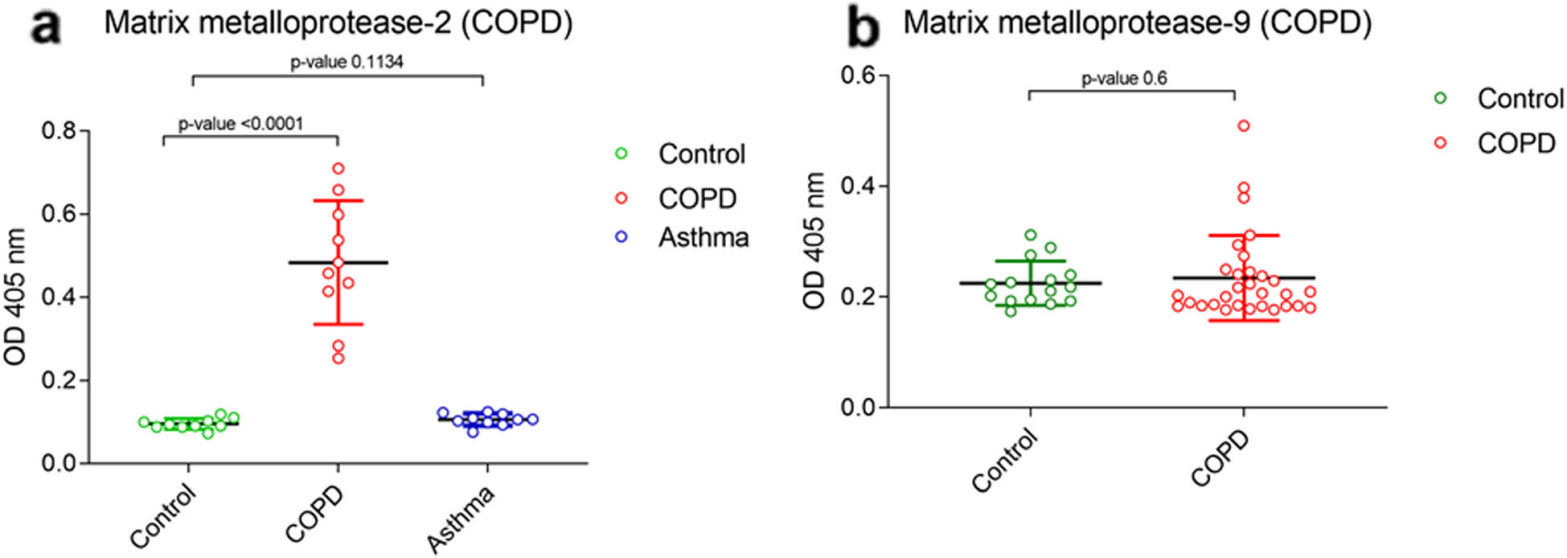
Qualitative analysis of serum MMP-2 in controls vs COPD vs Asthma patients and serum MMP-9 from controls and COPD patients. The p-values are calculated by unpaired t-test with 95% confidence interval using Graphpad prism 5.0 software

MMPs are the zinc/calcium-dependent endopeptidases that play crucial role in the extracellular matrix remodelling [21]. MMPs are crucial in pathogenesis of both respiratory diseases, COPD and Asthma; therefore, we attempted to assess crucial MMPs, which are distinct for COPD only. In the present study, the qualitative analysis of serum MMP-2 from equal number of subjects from three groups- controls, COPD and Asthma patients, revealed a significant elevation of serum MMP-2 in COPD patients and controls group [*p < 0.0001*; 95% CI] (Fig. 4a). Previously, the role of MMP-9 has been implicated in various cellular processes such as cellular migration and airway inflammatory responses in COPD [22] and Asthma [23]. However, no significant difference in serum MMP-9 was observed in controls and COPD patients [*p = 0.6*; 95% CI] (Fig. 4b). The quantitative analysis of serum MMP-2 in COPD patients estimated a significant increase with an average concentration of (0.71 ± 0.0647 μg/ml) as compared to the controls (0.05 ± 0.0083 μg/ml) (Table 1).

### Increase in ROS levels in COPD patients

**Fig. 5:**
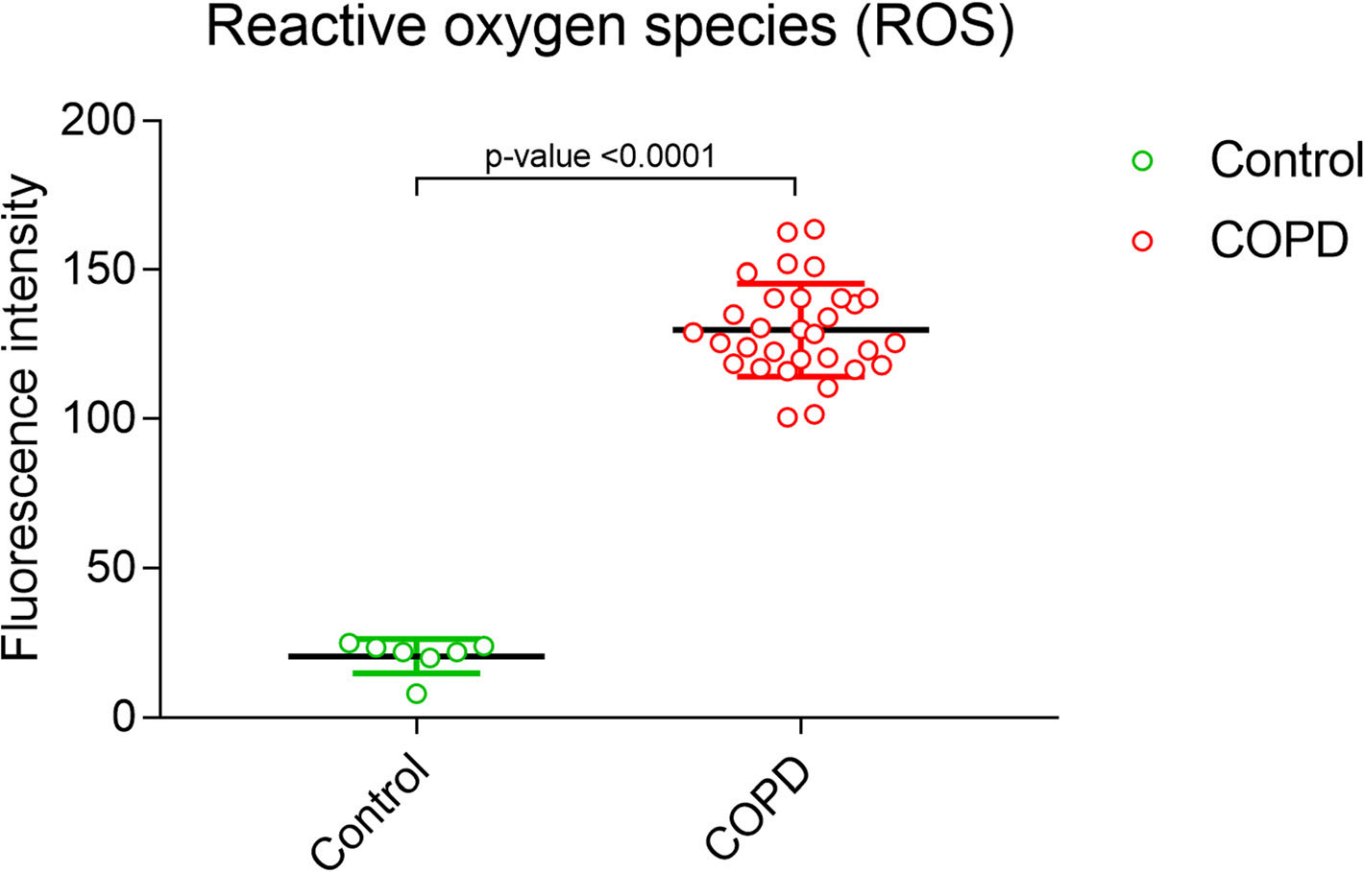
Measurement of reactive oxygen species (ROS) in controls and COPD patients. The p-values are calculated by unpaired t-test with 95% confidence interval using Graphpad prism 5.0 software

A key characteristic of COPD is the disruption of the oxidant/antioxidant balance due to generation of reactive oxygen species (ROS) from exogenous sources such as cigarette smoke, air pollutants or from endogenous sources viz. neutrophils and macrophages [24]. Therefore, the generation of ROS is a prominent indicator of the inflammatory reactions occurring in COPD. The present study estimated ROS from controls and COPD patients sera; a significantly elevated ROS in COPD patients vs controls indicated towards disruption of oxidant-antioxidanct balance [*p < 0.0001*; 95% CI] (Fig. 5).

### Mass spectrometric analysis of COPD proteome

The mass spectrometric analysis is an extremely sensitive technique and has become a method of choice for analysing the proteome of disease samples vs the controls. Signature proteins can be quickly identified from a relatively small sample volume. After the biochemical analysis of various serum proteases, we performed proteomics analysis of 7 COPD patients and 1 control. The proteomic analysis enabled us to identify differentially expressed proteins in COPD patients. Amongst, the differentially expressed proteins some of the proteins were in higher orders of expression as compared to the controls and vice-versa (Table 2). The major proteins which had a negative fold-change in COPD patients vs the controls were protease inhibitors- Preg. Zone protein, α-2 Macroglobulin (A2MG), Peptidase Inhibitor (PI16). The decreased levels of protease inhibitors strongly point towards an altered protease-antiprotease balance, as higher protease activities correlate well with decreased protease inhibitor concentrations in COPD. Another protein found to have negative fold change was Serotransferrin (TRFE_Human), which is also an important part of the defense against oxidative damage and also corroborated with the increased ROS levels in COPD patients. Interestingly, among the proteins with positive fold-change were proteases such as Carboxy peptidase B2 (CBPB2), Matrix Metalloprotease-2 (MMP-2) and Human Leukocyte Elastase (HLE). In our study, the positive fold-change represented the degradative processes as observed in COPD patients. Another protein (cytokine suppressor (SOCS-3)) was also identified with positive fold-change, which has been reported to be involved in the negative regulation of cytokines, correlating well with abrupt cytokine signaling in COPD.

**Table 2:** List of proteins with altered expression in COPD patients vs the controls, as per the MALDI sequencing analysis

S. No.	Name of the identified proteins	Fold change	Predicted function
1	Preg. Zone Protein (PZP)	−3.6	Protease inhibitor, able to inhibit all four classes of proteases
2	α-2 Macroglobulin(A2MG)	−2.5	Protease inhibitor, able to inhibit all four classes of proteases
3	Peptidase Inhibitor (PI16)	−2.3	Serine Protease Inhibitor
4	Serotransferrin TRFE_Human)	−2.0	To prevent oxidative damage
5	Cytokine suppressor (SOCS-3)	3.1	SOCS-3; Suppressor of cytokine signaling. SOCS3 involved in the negative regulation of cytokines.
6	Carboxy peptidase B2,(CBPB2)	2.4	Carboxy peptidase (cleave basic amino residues), plays a major role in the breakdown of the extracellular matrix
7	Matrix Metallo Protease −2 (MMP-II)	2.6	MMP-2 or Gelatinase A or type IV Collagenase, breakdown extracellular matrix
8	Human Leukocyte Elastase (HLE)	2.8	Serine Protease that hydrolyzes many proteins in addition to Elastin

## Discussion

COPD is the most common respiratory diseases and is characterized by various degradative processes, remodelling of the extracellular matrix (ECM) and oxidative damage in the lung environment. It is imperative to distinctly identify robust biomarkers for COPD, as many of the symptoms of COPD often overlap with other respiratory diseases such as Asthma. In thi study, we investigated various serum protease, which can be exploited as candidate biomarkers for COPD. Notably, serum NE in COPD have been implicated in multiple studies- altered ratio of serum NE (protease) and α-1 antitrypsin (A1AT) (antiprotease) have been shown to be directly correlated with the disease severity [7]; in vivo NE activity has been reported as a marker for cross-sectional COPD disease severity [25]. Although serum NE has consistently been argued as a preliminary biomarker of COPD, our study reports elevated serum NE in both the respiratory illnesses (COPD and Asthma). Therefore, questioning the distinctiveness of serum NE as a biomarker for COPD. Suppression of inflammatory responses by DPP-IV has been previously reported in tumor biology by inactivating the neuropeptides, peptide hormones and chemokines. The quantitative analysis of DPP-IV from our study also corroborated the decrease in serum DPP-IV concentrations as an indicator of COPD. Due to the versatile inflammatory responses, resulting in altered DPP-IV activity, its specific role in COPD as a biomarker would be challenging to validate.

Caspase-7 has been termed as an executioner caspase with implications in cell death and proteolysis. It has also been previously reported to be upregulated in case of acute brain tissue injury in rats, suggesting its role in neuronal cell death [26]. It is known that caspase-7 in association with caspase-12 has been linked to the endoplasmic reticulum pathway of apoptosis which is induced via stress, and further activates the effector caspase-3 [6]. The elevated caspase-7 (executioner caspase) could be responsible for the induction of inflammatory responses and cell death via apoptosis in COPD. An increased MMP-2 expression in the lung periphery has been reported to be associated with worsened lung function and increased emphysema, thus it is important for lung tissue remodelling and inflammation in COPD [27]. Corroborating the elevated MMP-2 in COPD, we report a significant increase in MMP-2 expression in COPD patients as compared to controls. The absence of lung tissue remodelling processes in Asthma as compared to COPD, also aligns well with our observation for nonsignificant difference in serum MMP-2 in controls and Asthma patients.

The mass spectrometric analysis of COPD proteome also identified positive fold-change in MMP-2 expression. We speculate that the difference in serum MMP-2 in COPD vs Asthma can be exploited as a differentiating biomarker between Asthma and COPD, along with other respiratory diseases in a larger cohort. The increased ROS in COPD patients is an indication of the elevated protease activities that results in upregulation of the cellular oxidative stress. Moreover, the increased ROS could also be correlated with the altered ionic balance and release of inflammatory cytokines which aid in the severity of the disease.

From the present study, following inferences have been made- 1) Serum NE cannot be used as distinctive biomarker of COPD, as we observed significantly higher serum NE in Asthma also; 2) decrease in DPP-IV could be due to suppression of inflammatory responses and hence does not specifically represent COPD signatures; 3) caspase-7, an executioner caspase which would have been recruited from multiple inflammatory signals, not specifically from COPD; 4) elevated ROS could also be a representation of higher protease activities and hence cannot be sourced alone from COPD and 5) increased MMP-2 expression, validated by ELISA as well as by mass spectrometric analysis, correlates well with emphysema in COPD, as well as in distinguishing Asthma from COPD.

## Conclusions

The dysregulation of proteases and anti-proteases in COPD has been reported previously in various studies. NE has been repeatedly shown to be a biomarker of COPD, but elevation of serum NE in both COPD and Asthma, can limit its specificity as a distinctive biomarker for COPD. Owing to the role of MMP-2 in extracellular remodeling processes in COPD alsone and correlating the increased expression in COPD, we speculate that MMP-2 can serve as distinctive biomarker for stable COPD. Moreover, the elevated serum MMP-2 have also been quantitatively estimated and further validated by the mass spectrometry data. Therefore our study concludes that MMP-2 should be validated as a candidate biomarker for COPD; further subjected to its rigorous validation by conducting large cohort studies.

## Data Availability

All the related data is presented in the manuscript. Information related to COPD patients can be obtained from the corresponding author on reasonable request.

## Abbreviations

COPD: Chronic pulmonary obstructive disease
NE: Neutrophil elastase
MMP-2: Matrix metalloprotease-2
MMP-9: Matrix metalloprotese-9
DPP-IV: Dipeptidyl peptidase-IV
ROS: Reactive oxygen species
